# Conducting a study to assess the long-term impacts of injury after 9/11: participation, recall, and description

**DOI:** 10.1186/s40621-019-0186-y

**Published:** 2019-03-18

**Authors:** Melanie H. Jacobson, Robert M. Brackbill, Patricia Frazier, Lisa M. Gargano

**Affiliations:** 1 World Trade Center Health Registry, Division of Epidemiology, New York City Department of Health and Mental Hygiene, 125 Worth Street, New York, NY 10013 USA; 2 Department of Psychology, University of Minnesota, Minneapolis, MN 55455 USA

**Keywords:** Injury, Longitudinal study, Disaster

## Abstract

**Background:**

The World Trade Center (WTC) attacks on September 11, 2001 (9/11) resulted in over 2700 fatalities and thousands injured. Injury on 9/11 has been identified as a risk factor for physical and mental health conditions, but the reasons for this are not well understood. In a population exposed to 9/11 and since followed, an in-depth study on the impacts of injury on 9/11 was conducted to identify factors that contribute to long-term functional issues. This report sought to examine factors influencing participation, participant recall of injury status over time, and determinants of injury severity.

**Methods:**

Enrollees from the World Trade Center Health Registry who completed all surveys between 2003 and 2016 and initially reported being injured (*N* = 2699) as well as a sample of non-injured (*N* = 2598) were considered to be eligible for the Health and Quality of Life 15 Years after 9/11 (HQoL) Study. Predictors of study non-participation and inconsistent recall of injury over time (i.e., discrepant reports) were identified through fitting log binomial models.

**Results:**

Participation rates were high overall (76.1%) and did not vary by initially reported injury status, although younger (vs. older), non-White (vs. White), and less educated (vs. more educated) enrollees were less likely to participate in the HQoL Study. Discrepant reporting of 9/11 injury status was much more common among enrollees who initially reported being injured on 9/11 (49.6%) compared with those who did not (7.3%). However, those who incurred more severe injuries on 9/11 were less likely to have discrepant reporting over time compared with those with more minor injuries (broken bone vs. sprain: risk ratio = 0.33, 95% Confidence Interval: 0.19, 0.57). Among those who consistently reported that they were injured on 9/11, most injuries occurred as a result of descending down stairs (31.5%) or by tripping and falling (19.9%); although being hit by a falling object was most often associated with high severity injuries (63.2%) compared with other modes of injury.

**Conclusions:**

These findings highlight the methodological issues involved in conducting a study on the long-term impact of injury more than a decade after the initial incident and may be relevant to future investigators. Factors affecting participation rates, such as demographic characteristics, and those related to discrepant reporting over time, such as injury severity, may affect both the internal and external validity of studies examining the long-term impact of injury.

**Electronic supplementary material:**

The online version of this article (10.1186/s40621-019-0186-y) contains supplementary material, which is available to authorized users.

## Introduction

The impacts on health from physical injuries sustained from natural or human-made disasters are complex due to the simultaneous experience of psychological trauma, stress, and physical wounds. The terrorist attacks on the World Trade Center (WTC) on September 11, 2001 (9/11) resulted in thousands seriously injured in conjunction with experiencing a substantial burden of new or exacerbated mental health morbidities. The World Trade Center Health Registry (Registry) is a large prospective cohort study established in order to study and track the health outcomes of people exposed to this disaster and presents an opportunity to examine the long-term impacts of simultaneous injury and trauma.

A significant body of literature has documented an association between traumatic injury and long-term health outcomes, such as posttraumatic stress disorder (PTSD) and poor self-rated health (Baragaba et al. 2016; Toft et al. 2010; North et al. 1999; Van den Berg et al. 2009). Likewise, several Registry studies have identified injury on 9/11 as a risk factor for both mental (Brackbill et al. 2009) and physical health conditions (Brackbill et al. 2014; Alper et al. 2017). Furthermore, a recent qualitative study examining the experiences of people who were injured on 9/11 documented a major diminution of quality of life as represented by physical and functional impairments, economic difficulties, and social isolation (Gargano et al. 2016). Motivated by these observations, an in-depth study on the long-term impacts of injury on 9/11 was conducted in order to identify factors that contribute to long-term functional issues and provide guidance for ameliorating these outcomes through intervention.

Although several previous Registry studies have documented the proportion of individuals who were injured and the factors that influenced the risk of injury (Farfel et al. 2008; Brackbill et al. 2006), no studies have examined how these injuries occurred or assessed potential predictors of injury severity. In addition, when researchers working on longitudinal studies that span several years then conduct nested sub-studies on more specific topic areas, the potential for selective participation (Mein et al. 2012; Weisskopf et al. 2015) and discrepant reporting arises (or may be exacerbated) (Beckett et al. 2000, 2001), but is often ignored or not reported in published studies. This study aimed to address these gaps in the literature.

This study had two overall objectives using data from the in-depth study on the long-term impacts of injury on 9/11. The first was to describe the complex issues related to conducting a sub-study nested within a longitudinal cohort study several years after the sentinel event of interest. Specifically, we sought to investigate factors influencing participation overall as well as any differences in participation and factors influencing participation between the injured and non-injured (i.e., comparison group). In addition, we compared participant recall of injury status as reported soon after 9/11 and again 15 years later and explored its potential impact in epidemiologic studies. The second overall objective was to characterize determinants of injury severity and describe how injuries were sustained on 9/11.

## Methods

### Original World Trade Center Health Registry study

#### Population

The Registry is a prospective cohort study of first responders, residents, area workers, and others who were present in downtown Manhattan on September 11, 2001. In 2003–04, 71,426 individuals were enrolled into the study and completed a baseline questionnaire (Wave 1), which was followed in subsequent years by Wave 2 (2006–07), Wave 3 (2011–12), and Wave 4 (2015–2016). Further details of this study have been published previously (Brackbill et al. 2009; Farfel et al. 2008). The Registry protocol was approved by the Institutional Review Boards of the Centers for Disease Control and Prevention and the New York City Department of Health and Mental Hygiene.

#### Measures

Wave questionnaires contained details on demographic factors such as race and ethnicity, household income, education, marital status, and employment status, as well as questions about specific health conditions and self-rated health. In addition, on Waves 1 and 2, questions were asked about various exposures on 9/11 and in the following days and weeks, such as the traumatic experiences on 9/11. Based on work by Adams and Boscarino (Adams and Boscarino 2005), Brackbill et al. derived a composite score consisting of 11 questions about traumatic experiences such as: being in the North or South WTC towers at the time of the attack; witnessing three or more events (seeing planes hit the buildings, people fall or jump from buildings, people injured, or people running); fear of being injured or killed; and having a relative killed on 9–11 (Brackbill et al. 2013). For this study, this score was adapted by removing the component of whether enrollees were injured. These items were summed (range = 0–10) and the score was then categorized as none/low (0–1 exposures), medium (2–3), high (4–5), and very high (≥ 6).

PTSD symptoms were assessed at each wave (Waves 1–4) using the stressor-specific PTSD Checklist (PCL)-17 (Blanchard et al. 1996; Ruggiero et al. 2003; Weathers et al. 1994), which contains direct references to the events of 9/11 in the re-experiencing and avoidance domains. The PCL is a self-administered questionnaire that queries the severity of PTSD symptoms based on *DSM-IV* criteria (American Psychiatric Association, n.d.) in three domains: re-experiencing, avoidance, and hyperarousal. Enrollees rated the degree to which these symptoms bothered them over the last 30 days, ranging from 1 = *not at all* to 5 = *extremely*, and the scores from the 17 items were summed. Total scores ≥44 were considered to be indicative of probable PTSD (hereafter referred to as PTSD) (Blanchard et al. 1996). PTSD status was summarized across time as ever (scores ≥44 on at least one wave) and never (scores < 44 at all waves).

#### Reporting on injury on 9/11 status

At Wave 1, enrollees were asked whether they suffered an injury on 9/11 as a result of the WTC terrorist attacks. Specifically, they were asked if they suffered any of the following: cut, abrasion, or puncture wound; eye injury or irritation; sprain or strain; burn; broken bone, fracture, or dislocation; concussion, head injury, or knocked out by being hit on the head; or any other type of injury.

### In-depth study on injury

#### Population

**Fig. 1:**
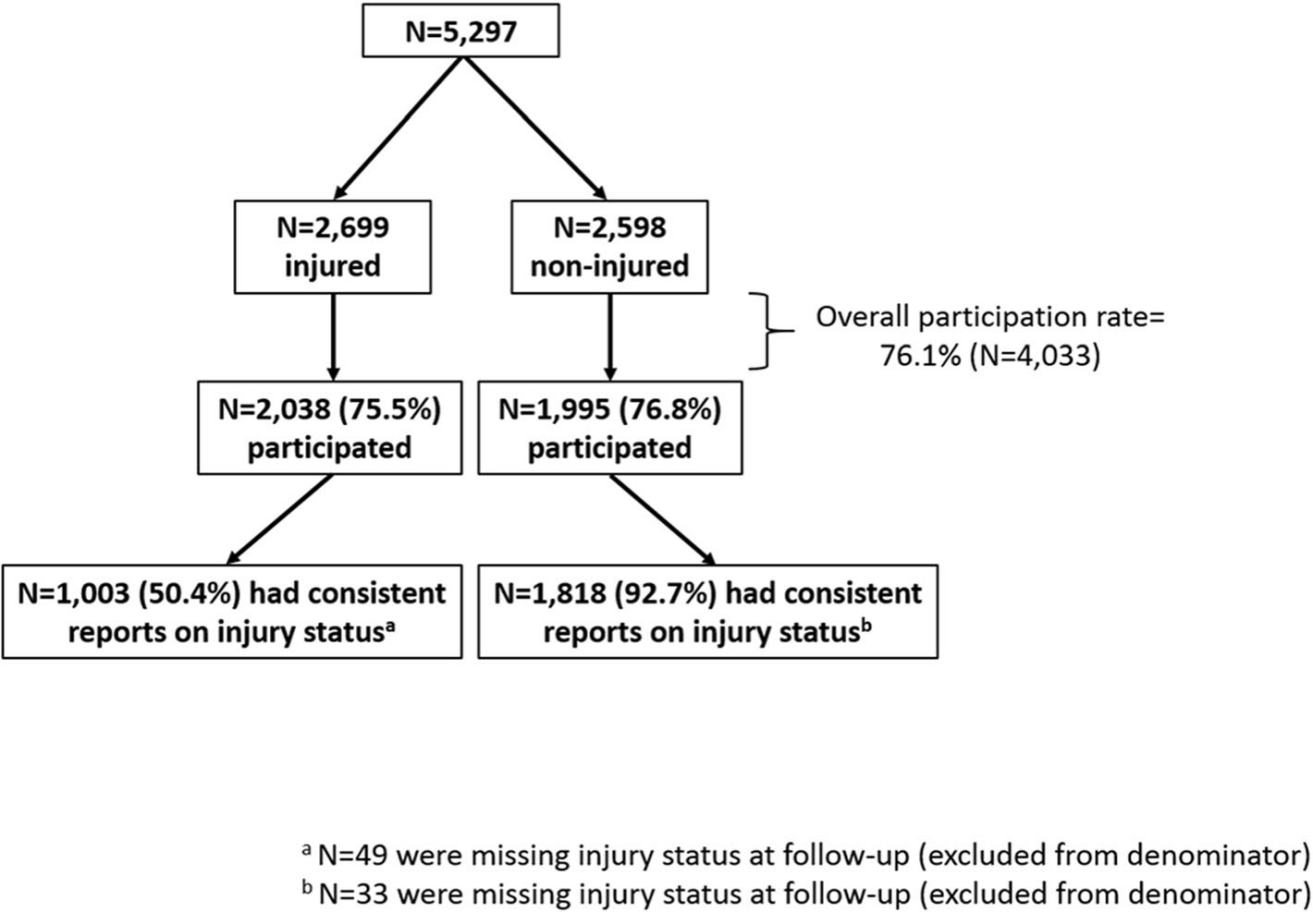
Flowchart of study participation, Health and Quality of Life 15 Years after 9/11 Study

To be eligible for the in-depth study, enrollees had to complete all four survey Waves, be ≥18 at Wave 1, speak English, and have reported being south of Chambers Street in Manhattan, NY on the morning of September 11, 2001 on Wave 1. Injured and non-injured individuals who were eligible as described above were then selected based on their responses to the Wave 1 questions about whether they were injured on 9/11. To be eligible for the in-depth study as ‘injured’, participants had to endorse at least one injury other than eye or other on the Wave 1 questionnaire; participants who did not endorse any of the injury types on the Wave 1 questionnaire were considered to be eligible as ‘non-injured’. All those who were eligible as injured were invited to participate, and a simple random sample of a similar size to that of the injured of those who were eligible as non-injured were invited to participate (Fig. 1). In an effort to avoid differential participation by injury group, the questionnaire was entitled “The Health and Quality of Life 15 Years after 9/11 Study” (HQoL Study). The study protocol was approved by the NYC Department of Health and Mental Hygiene’s Institutional Review Board.

#### Questionnaire administration

Invitations to participate in the HQoL study were sent in early March 2017 by postal mail or e-mail, if it was available. Approximately 10 days later, personalized e-mails with links to the online questionnaire were sent to enrollees for whom there was a known e-mail address (90.7%); personalized letters with a paper questionnaire were sent to the homes of those for whom there was no e-mail address (9.3%). For those with e-mail addresses, e-mail reminders were sent weekly thereafter. For those with no e-mail, postcard reminders were sent monthly, with a total of three sent over 3 months. In early May 2017, paper questionnaires were sent to the homes of all those who had not completed the questionnaire by that time, including those who did and did not have e-mail addresses. This was done again in mid-June 2017. In addition, two smaller mailings of the paper questionnaires were done, in mid-April and at the end of May, for those questionnaires that were returned with a forwarding address as well as those who called to request a paper questionnaire. Incentives were not initially offered, but to spur more participation toward the end of data collection, $10 gift cards were offered for those who completed the questionnaire starting in mid-June. The deadline for online or paper questionnaire completion was July 14, 2017.

#### Measures

The questionnaire was estimated to take about 15–30 min to complete and spanned several subject areas. First, with regard to assessing injury on 9/11 status, enrollees were asked if they were injured on 9/11, how it happened (i.e., hit by a falling object; tripped and fell; etc.); and what types of medical intervention or support were sought in the weeks after 9/11 to treat the most serious injury sustained. Additional questions were asked on topics such as mental health and others not used in this particular investigation: functional status, quality of life, somatic symptoms, and posttraumatic growth. Questions were adapted from the *Behavioral Risk Factor Surveillance System* (Moriarty et al. 2003), 12-item short form survey (Ware Jr et al. 1996), Patient Health Questionnaire (Kroenke et al. 2009; Löwe et al. 2010), National Institute of Standards and Technology Questionnaire on Emergency Evacuation Procedures (Kuligowski and Hoskins 2011), Somatic Symptom Scale-8 (Gierk et al. 2014), the Posttraumatic Growth Inventory (Tedeschi and Calhoun 1996), and the 6-item De Jong Gierveld Loneliness Scale (Gierveld and Tilburg 2006); among others.

### Statistical analysis

In order to evaluate what factors influenced participation in the HQoL Study, those who participated were compared to those who did not participate by injury on 9/11 status as reported at Wave 1, sociodemographic characteristics, WTC-related exposures, PTSD history, and self-rated health. We also assessed whether the factors that may have influenced participation differed between the injured and non-injured groups by stratifying by injury on 9/11 status as reported at Wave 1 in subsequent analyses. Multivariable log binomial models were fit to identify predictors of non-participation, which yielded adjusted risk ratios (aRR) and 95% confidence intervals (CI). Models controlled for injury on 9/11 status, sociodemographic characteristics (age, sex, race/ethnicity, education, marital status, income, and employment status), Registry eligibility group, WTC-related exposures, PTSD history, and self-rated health. Among those who participated, we then explored the extent of discrepant reports of injury on 9/11 status by comparing Wave 1 and HQoL Study questionnaire responses. A discrepant response was defined as either a report of injury on 9/11 at Wave 1 but no injury on 9/11 reported on the HQoL survey, or a report of no injury on 9/11 at Wave 1 but a report of injury on 9/11 on the HQoL survey. We identified predictors of discrepant reports by comparing frequencies across strata of injury on 9/11 status as reported at Wave 1, sociodemographic characteristics, WTC-related exposures, and PTSD history and by fitting log binomial models controlling for these factors. Next, similar to our analysis examining predictors of participation, in order to assess whether these predictors varied by injury on 9/11 status as specified at Wave 1, we stratified the analysis by injury on 9/11 status as reported at Wave 1. Among those who specified that they were injured at Wave 1, we added injury type (i.e., sprain, broken bone, etc.) and injury type count (i.e., 1–5) to the model. Finally, we evaluated how errors in recall could affect a hypothetical study that did not have this information over time. We conducted an illustrative analysis estimating the association between injury on 9/11 and PTSD at Wave 4 (i.e., a score of ≥44 on the PCL) in three log binomial models with different ‘exposed’ and ‘unexposed’ groups. First, we fit a model comparing those who reported being injured at Wave 1 vs. those who did not among those who completed the HQoL study. Second, we fit a model comparing those who reported being injured at the HQoL survey vs. those who did not. Third, we fit a model comparing those who reported being injured at both surveys vs. those who reported not being injured at both surveys. All models controlled for age at 9/11, sex, race/ethnicity, employment status on 9/11, and Registry eligibility group.

Lastly, among those who consistently reported that they were injured on 9/11 (i.e., they reported it at Wave 1 and on the HQoL Study questionnaire), we examined injury severity in relation to sociodemographic characteristics, 9/11-related exposures, and how the injury was sustained. For those with multiple injuries, injury severity referred to the most serious injury received on 9/11. Injury severity was defined by the degree of medical intervention sought after the injury and was operationalized as follows: high severity included those who sought the most invasive treatments or used the most intense therapeutic measures, such as using a wheelchair, presenting at the emergency department, and/or having surgeries; medium severity were those that sought only supportive or rehabilitative measures such as rest and physical therapy; and low severity were those with superficial injuries that did not require any intervention or care. Multinomial logistic regression models were fit to identify determinants of injury severity, yielding adjusted odds ratios (aOR) and 95% CI. Models controlled for how injury occurred, age at 9/11, sex, race/ethnicity, employment status on 9/11, Registry eligibility group, and WTC-related exposures. Finally, for rescue and recovery workers, a separate model was fit excluding the term for eligibility group and with the following terms in addition: date of arrival and number of days worked at the WTC site.

## Results

### Study participation

Among those who completed the questionnaire, 62.3% completed it online and 37.7% on paper. Among those initially invited to participate by a paper mailing (9.3%), 99% completed it on paper and 1% completed it online (by calling the Registry and asking for a link to complete it online). Among those initially invited by an e-mail invitation with a link to the online questionnaire, 68.2% completed it online, and 31.8% completed it on paper, either by calling to request a paper copy or completing one after it arrived at their home during the later stages of recruitment (i.e., in May and June 2017). Participation rates did not markedly vary by mode of initial invitation (71.7% among paper invites and 76.8% among e-mail invites).

A total of 5297 eligible individuals were invited to participate in the HQoL Study (Fig. 1), with an approximately equal number of injured and non-injured. The overall participation rate was 76.1% and did not significantly differ by injury status (75.5% of injured participated vs. 76.8% of non-injured, risk ratio (RR) = 1.02 (95% Confidence Interval (CI): 0.90, 1.15)). Table 1 presents the participation rate by injury status, social and demographic characteristics, WTC-related exposures, PTSD history, and other factors. Age was positively associated with participation such that older individuals were more likely to participate compared with younger individuals. For example, compared with those aged 31–49 years at study invitation, those aged 70–94 years were less likely to *not* participate (i.e., more likely to participate, aRR = 0.61, 95% CI: 0.48, 0.78). Education level was also positively associated with participation, but participation rates did not vary significantly across income strata. Whites had greater participation rates (78.1%) compared with Blacks (72.5%), Latinos (70.0%), Asians (72.6%), and those of other race (68.1%). WTC-related exposure and a history of PTSD were not significantly related to participation, but perceived overall health status was positively associated with participation. For example, those who rated their health as excellent were less likely to *not* participate (i.e., more likely to participate) compared with those who rated their health as poor (RR = 0.74, 95% CI: 0.55, 0.98). Factors related to participation did not vary by injury on 9/11 status as assessed at Wave 1 (data not shown).

**Table 1:** Participation in the Health and Quality of Life 15 Years after 9/11 Study by demographic characteristics, injury, and health history, and World Trade Center (WTC) exposures

	Participated (*N* = 4033, 76.1%)	Did not participate (1264, 23.9%)	
	N (%)	N (%)	aRR^a^ (95% CI)
Injury status as reported at Wave 1			
Injured	2038 (75.5)	661 (24.5)	1.02 (0.90, 1.15)
Non-injured	1995 (76.8)	603 (23.2)	1.00 (Reference)
Age at time of study invitation (years)			
31–49	928 (69.8)	402 (30.2)	1.00 (Reference)
50–59	1308 (76.2)	408 (23.8)	0.74 (0.65, 0.85)
60–69	1298 (79.7)	330 (20.3)	0.67 (0.58, 0.78)
70–94	499 (80.1)	124 (19.9)	0.61 (0.48, 0.78)
Sex			
Men	2311 (77.7)	664 (22.3)	1.00 (Reference)
Women	1722 (74.2)	600 (25.8)	1.06 (0.94, 1.20)
Race/ Ethnicity			
White	2963 (78.1)	829 (21.9)	1.00 (Reference)
Black	401 (72.5)	152 (27.5)	1.17 (0.98, 1.40)
Latino	399 (70.0)	171 (30.0)	1.19 (1.01, 1.40)
Asian	159 (72.6)	60 (27.4)	1.15 (0.90, 1.48)
Other race	111 (68.1)	52 (31.9)	1.38 (1.07, 1.78)
Education at Wave 4			
≤ High school/ GED	434 (73.3)	158 (26.7)	1.00 (Reference)
Some college	1128 (75.5)	366 (24.5)	0.83 (0.70, 0.99)
College	1287 (77.2)	381 (22.8)	0.77 (0.64, 0.92)
Graduate degree	1152 (78.2)	322 (21.8)	0.72 (0.60, 0.88)
Marital Status at Wave 4			
Married/cohabitating	2742 (77.6)	792 (22.4)	1.00 (Reference)
Divorced	508 (71.5)	202 (28.5)	1.23 (1.06, 1.43)
Widowed	156 (77.6)	45 (22.4)	1.01 (0.74, 1.39)
Never married	583 (75.8)	186 (24.2)	1.02 (0.87, 1.20)
Income at Wave 4			
≤ $50,000	696 (73.9)	246 (26.1)	1.00 (Reference)
$50,000–$150,000	1912 (77.4)	559 (22.6)	0.93 (0.79, 1.08)
≥ $150,000	1194 (76.0)	378 (24.0)	1.08 (0.90, 1.30)
Employment status at Wave 4			
Employed	2418 (75.0)	808 (25.0)	1.00 (Reference)
Retired	1050 (80.6)	252 (19.4)	0.91 (0.77, 1.07)
Student/Homemaker	63 (68.5)	29 (31.5)	1.23 (0.89, 1.69)
Unemployed due to health reasons	289 (75.5)	94 (24.5)	0.80 (0.63, 1.00)
Unemployed due to other reasons	146 (78.1)	41 (21.9)	0.81 (0.59, 1.11)
Eligibility group			
Rescue and recovery worker	1114 (76.9)	334 (23.1)	1.00 (Reference)
Lower Manhattan resident	399 (70.9)	164 (29.1)	1.22 (0.99, 1.50)
Lower Manhattan area worker/passerby	2520 (76.7)	766 (23.3)	1.03 (0.89, 1.19)
WTC exposure score			
None/low	682 (78.3)	189 (21.7)	1.00 (Reference)
Medium	1585 (76.0)	501 (24.0)	1.05 (0.89, 1.23)
High	1373 (75.4)	448 (24.6)	1.08 (0.90, 1.29)
Very high	393 (75.7)	126 (24.3)	0.97 (0.76, 1.24)
Ever-PTSD^b^			
No	2269 (77.7)	650 (22.3)	1.00 (Reference)
Yes	1519 (75.3)	498 (24.7)	0.96 (0.84, 1.09)
Self-rated health at Wave 4			
Excellent	347 (76.9)	104 (23.1)	0.74 (0.55, 0.98)
Very Good	1113 (79.1)	294 (20.9)	0.64 (0.50, 0.82)
Good	1485 (77.2)	439 (22.8)	0.74 (0.59, 0.92)
Fair	842 (73.9)	298 (26.1)	0.85 (0.68, 1.06)
Poor	209 (70.4)	88 (29.6)	1.00 (Reference)

^a^ Risk ratio models the outcome as *not* participating. Model controls for all factors listed in the table

^b^ Ever had a PCL score ≥ 44 on Wave 1, Wave 2, Wave 3, and/or Wave 4

Among those that participated in the HQoL study and reported injury status consistently over time, 35.6% were injured on 9/11 and 64.4% were not (Additional file 1: Table S1 and Fig. 1). Overall, the sample was predominately male (56.3%), White (74.5%), with a college degree or more (64.3%), and married (64.7%). About a quarter were rescue and recovery workers (23.5%), and most experienced a substantial degree of WTC-related exposures (80.8% had composite scores of medium or greater). Finally, about a third of the study sample had a history of PTSD symptoms above a clinical threshold. Those who were injured were more likely to be middle aged (35–49 years) than those who were not injured (57.6% vs. 44.9%, respectively). Other differences across demographic strata were also apparent: those who were injured were more likely to be male (vs. female); Black or Hispanic (vs. White or Asian); have less education than a college degree (vs. with a college education or higher); and have incomes ≤$50,000 (vs. >$50,000) compared with those who were not injured. Lastly, those were injured were more likely to have been rescue and recovery workers (vs. lower Manhattan community members); have high WTC-related exposures (vs. low); and have a history of PTSD (vs. no history) compared with those who were not injured.

### Recall of injury over time

Among the 4033 enrollees who participated in the HQoL Study, 28.6% had inconsistent reports of injury status on 9/11 between Wave 1 and the HQoL Study. However, those who specified that they were injured at Wave 1 were much more likely to have discrepant reports at follow-up (49.6%, i.e., thus reporting that they were *not* injured on 9/11 on the HQoL survey) compared with those who reported that they were not injured on 9/11 at Wave 1 (7.3%) (RR = 9.61, 95% CI: 7.98, 11.57).

Overall, predictors of discrepant reports varied by Wave 1 injury status (Table 2). Among those who reported at Wave 1 that they were injured on 9/11, those with more severe injuries as well as those with several injuries were less likely to have discrepant reports at follow-up (i.e., reported injury on 9/11 at Wave 1 but did *not* report it again at the time of the HQoL survey) compared with those with more superficial injuries and just one injury type, respectively. For example, those who reported broken bones compared with sprains (RR = 0.33, 95% CI: 0.19, 0.57) and those with two or more injuries compared with one (RR = 0.60, 95% CI: 0.51, 0.69) were less likely to have discrepant reports. Other predictors of discrepant reports among those who reported injury at Wave 1 included marital status, unemployment, WTC exposure scores, and PTSD history. Specifically, those who were divorced (vs. married), unemployed due to health reasons (vs. employed), had higher WTC exposure scores, and with a history of PTSD (vs. no PTSD) were less likely to have discrepant reports.

**Table 2:** Demographics, World Trade Center (WTC) exposures, and injury and health history by consistency on injury status between World Trade Center Health Registry Wave 1 and the Health and Quality of Life 15 Years after 9/11 Study, stratified by injury status at Wave 1

	Injured at Wave 1			Not injured at Wave 1		
	Consistent (*N* = 1003, 50.4%)	Discrepant (*N* = 986, 49.6%)		Consistent (*N* = 1818, 92.7%)	Discrepant (*N* = 144, 7.3%)	
	N (%)	N (%)	aRR^a,b^ (95% CI)	N (%)	N (%)	aRR^a,c^ (95% CI)
Age at time of study invitation (years)						
31–49	181 (46.1)	212 (53.9)	1.00 (Reference)	479 (95.0)	25 (5.0)	1.00 (Reference)
50–59	380 (53.9)	325 (46.1)	0.94 (0.85, 1.05)	529 (92.6)	42 (7.4)	1.24 (0.73, 2.10)
60–69	333 (50.5)	327 (49.5)	0.95 (0.84, 1.07)	568 (91.0)	56 (9.0)	1.46 (0.84, 2.55)
70–94	109 (47.2)	122 (52.8)	1.02 (0.87, 1.21)	242 (92.0)	21 (8.0)	2.15 (1.07, 4.34)
Sex						
Men	614 (51.0)	591 (49.0)	1.00 (Reference)	975 (92.2)	83 (7.8)	1.00 (Reference)
Women	389 (49.6)	395 (50.4)	1.03 (0.94, 1.13)	843 (93.3)	61 (6.7)	0.70 (0.47, 1.04)
Race/ Ethnicity						
White	715 (50.0)	715 (50.0)	1.00 (Reference)	1386 (93.6)	95 (6.4)	1.00 (Reference)
Black	112 (51.9)	104 (48.1)	0.96 (0.83, 1.11)	155 (89.6)	18 (10.4)	1.38 (0.81, 2.33)
Latino	117 (54.2)	99 (45.8)	0.94 (0.82, 1.09)	150 (87.7)	21 (12.3)	1.15 (0.67, 1.98)
Asian	31 (44.9)	38 (55.1)	1.11 (0.91, 1.37)	78 (90.7)	8 (9.3)	1.70 (0.83, 3.47)
Other race	28 (48.3)	30 (51.7)	1.03 (0.79, 1.34)	49 (96.1)	2 (3.9)	0.58 (0.15, 2.21)
Education at Wave 4						
≤ High school/ GED	137 (53.7)	118 (46.3)	1.00 (Reference)	150 (89.8)	17 (10.2)	1.00 (Reference)
Some college	365 (51.1)	349 (48.9)	1.03 (0.89, 1.18)	340 (88.3)	45 (11.7)	1.77 (0.89, 3.50)
College	294 (51.5)	277 (48.5)	0.98 (0.85, 1.13)	641 (93.4)	45 (6.6)	1.47 (0.73, 2.97)
Graduate degree	198 (45.9)	233 (54.1)	1.05 (0.90, 1.21)	675 (95.1)	35 (4.9)	1.49 (0.72, 3.09)
Marital Status at Wave 4						
Married/cohabitating	624 (47.6)	686 (52.4)	1.00 (Reference)	1285 (93.8)	85 (6.2)	1.00 (Reference)
Divorced	182 (61.3)	115 (38.7)	0.76 (0.65, 0.89)	178 (87.3)	26 (12.7)	1.63 (1.03, 2.56)
Widowed	50 (54.3)	42 (45.7)	0.90 (0.73, 1.11)	56 (91.8)	5 (8.2)	1.00 (0.41, 2.42)
Never married	128 (49.4)	131 (50.6)	0.98 (0.87, 1.10)	291 (92.4)	24 (7.6)	1.14 (0.69, 1.90)
Income at Wave 4						
≤ $50,000	250 (61.4)	157 (38.6)	1.00 (Reference)	239 (87.9)	33 (12.1)	1.00 (Reference)
$50,000–$150,000	453 (47.6)	499 (52.4)	1.02 (0.89, 1.17)	842 (91.8)	75 (8.2)	0.88 (0.56, 1.39)
≥ $150,000	236 (46.9)	267 (53.1)	0.93 (0.79, 1.10)	647 (96.3)	25 (3.7)	0.60 (0.32, 1.13)
Employment status at Wave 4						
Employed	492 (45.5)	589 (54.5)	1.00 (Reference)	1202 (94.1)	76 (5.9)	1.00 (Reference)
Retired	282 (50.2)	280 (49.8)	0.90 (0.80, 1.01)	436 (90.8)	44 (9.2)	1.04 (0.65, 1.66)
Student/Homemaker	6 (40.0)	9 (60.0)	1.02 (0.89, 1.16)	43 (93.5)	3 (6.5)	2.05 (0.52, 8.06)
Unemployed due to health reasons	168 (77.1)	50 (22.9)	0.54 (0.41, 0.71)	51 (78.5)	14 (21.5)	1.29 (0.73, 2.28)
Unemployed due to other reasons	31 (42.5)	42 (57.5)	1.15 (0.98, 1.36)	64 (94.1)	4 (5.9)	0.81 (0.31, 2.10)
Eligibility group						
Rescue and recovery worker	423 (51.3)	401 (48.7)	1.00 (Reference)	241 (89.3)	29 (10.7)	1.00 (Reference)
Lower Manhattan resident	66 (51.2)	63 (48.8)	1.00 (0.84, 1.21)	252 (95.1)	13 (4.9)	0.46 (0.22, 0.97)
Lower Manhattan area worker/passerby	514 (49.6)	522 (50.4)	0.92 (0.84, 1.01)	1325 (92.9)	102 (7.1)	0.79 (0.51, 1.23)
WTC exposure score						
None/low	25 (24.3)	78 (75.7)	1.00 (Reference)	537 (95.0)	28 (5.0)	1.00 (Reference)
Medium	228 (37.9)	373 (62.1)	0.87 (0.78, 0.97)	896 (94.1)	56 (5.9)	1.15 (0.70, 1.89)
High	491 (52.2)	449 (47.8)	0.77 (0.69, 0.87)	355 (87.2)	52 (12.8)	1.80 (1.08, 3.02)
Very high	259 (75.1)	86 (24.9)	0.47 (0.38, 0.59)	30 (78.9)	8 (21.1)	2.58 (1.13, 5.88)
Ever-PTSD^d^						
No	314 (38.2)	508 (61.8)	1.00 (Reference)	1357 (96.1)	55 (3.9)	1.00 (Reference)
Yes	641 (61.0)	410 (39.0)	0.79 (0.72, 0.87)	354 (82.5)	75 (17.5)	3.32 (2.25, 4.90)
Types of injuries specified at Wave 1						
Cut	618 (56.5)	476 (43.5)	0.81 (0.75, 0.88)			
Broken bone	102 (87.9)	14 (12.1)	0.33 (0.19, 0.57)			
Burn	149 (64.5)	82 (35.5)	0.76 (0.64, 0.90)			
Concussion	81 (94.2)	5 (5.8)	0.22 (0.09, 0.50)			
Sprain	581 (50.3)	575 (49.7)	1.00 (Reference)			
Injury type^e^ count as measured at Wave 1						
1	624 (42.7)	836 (57.3)	1.00 (Reference)			
2^f^	270 (66.2)	138 (33.8)	0.60 (0.51, 0.69)			
3	75 (89.3)	9 (10.7)				
4	28 (93.3)	2 (6.7)				
5	6 (85.7)	1 (14.3)				

^a^Risk ratio models the outcome as being *discordant* on injury status

^b^Model fit among those who specified they were injured at Wave 1 includes all covariates listed in this column

^c^Model fit among those who specified they were *not* injured at Wave 1 includes all covariates listed in this column

^d^Ever had a PCL score ≥ 44 on Wave 1, Wave 2, Wave 3, and/or Wave 4

^e^Count of the following injury types: cut, broken bone, burn, concussion, sprain

^f^Risk ratio represents comparison between those who had two or more injuries versus those who had just one

In contrast, among those who reported *not* being injured at Wave 1, participants who were older at the time of study invitation were more likely to have discrepant reports (i.e., reported no injury on 9/11 at Wave 1 but *did* reportinjury on 9/11 at the time of the HQoL survey) compared with younger participants (70–94 years vs. 31–49 years, 2.15, 95% CI, 1.07, 4.34). In addition, marital status, WTC exposure scores, and PTSD history were associated with an increased likelihood of discrepant reports. Specifically, enrollees who were divorced (vs. married) were more likely to have discrepant reports (RR = 1.63, 95% CI: 1.03, 2.56) as well as those who had high or very high scores on the World Trade Center exposure score scale (vs. none or low scores) and those with a history of PTSD compared to those with no PTSD (RR = 3.32, 95% CI: 2.25, 4.90). Lastly, women (vs. men) and lower Manhattan residents (vs. rescue and recovery workers) were *less* likely to have discrepant reports.

The results of the illustrative analysis comparing three differently defined exposed and unexposed groups are shown in Additional file 1: Table S2. Injury on 9/11 was associated with PTSD status at Wave 4 in all models, and results were similar when injury on 9/11 status was based on Wave 1 or the HQoL survey. However, when injury on 9/11 status was defined as reporting it consistently at both surveys, the estimate was 69–78% greater than estimates from models where injury status was based on Wave 1 or the HQoL survey.

### Injury description

A total of 1003 individuals consistently reported that they were injured on 9/11 on both the Wave 1 survey and the HQoL Study questionnaire. Table 3 shows the distribution of injury severity as defined by the degree of medical intervention sought after injury, as well as the determinants of injury severity. High and medium severity injuries constituted the majority of all injury (40.1 and 47.3%, respectively), with only 12.7% experiencing low severity injuries. The most common way of sustaining any injury on 9/11 was by descending down stairs (31.5%), followed by tripping and falling (19.9%), and coming into contact with something hot, such as fire or ashes (13.3%). However, the modes of injury occurrence that were most often related to high severity injuries were being hit by a falling object (63.2%) and being covered by dust, debris, ash, and/or asbestos (60.0%), although only 17 individuals (1.7%) reported the latter situation as the cause of their most serious injury. However, in multivariable analyses, none of these individual modes of injury were statistically significantly associated with severity (Table 4).

**Table 3:** Injury severity by injury circumstances, demographic characteristics, eligibility group, and World Trade Center (WTC) exposures, Health and Quality of Life 15 Years after 9/11 Study

	Total injured^a^ *N* = 1003	Severity index			
		Low (*N* = 120 (12.7%))	Medium (*N* = 448 (47.3%))	High (*N* = 380 (40.1%))	Missing (*N* = 55)
Characteristic	N (%)	N (%)	N (%)	N (%)	N
How injury occurred (among injured)^b^					
Hit by a falling object	98 (9.8)	7 (7.4)	28 (29.5)	60 (63.2)	3
Trip and fell	200 (19.9)	18 (9.2)	104 (53.3)	73 (37.4)	5
Hit head on object	83 (8.3)	18 (23.4)	32 (41.6)	27 (35.1)	6
Came into contact with something hot	133 (13.3)	9 (7.0)	73 (57.0)	46 (35.9)	5
Descending down stairs	316 (31.5)	27 (9.3)	145 (49.8)	119 (40.9)	25
Trapped, buried, or crushed	58 (5.8)	3 (5.6)	29 (53.7)	22 (40.7)	4
Covered by dust/debris/ash/asbestos	17 (1.7)	2 (13.3)	4 (26.7)	9 (60.0)	2
Unknown or unsure	98 (9.8)	36 (38.7)	33 (35.5)	24 (25.8)	5
Injury type count^c^ as measured at Wave 1					
1	624 (62.2)	85 (14.5)	316 (53.7)	187 (31.8)	0
2	270 (26.9)	28 (10.9)	110 (43.0)	118 (46.1)	0
3	75 (7.5)	7 (10.0)	16 (22.9)	47 (67.1)	0
4	28 (2.8)	0 (0.0)	6 (21.4)	22 (78.6)	0
5	6 (0.6)	0 (0.0)	0 (0.0)	6 (100.0)	0
Age at 9/11 (years)					
16–34	181 (18.1)	15 (8.8)	85 (49.7)	71 (41.5)	10
35–49	578 (57.6)	67 (12.3)	262 (48.1)	216 (39.6)	33
≥ 50	244 (24.3)	38 (16.4)	101 (43.5)	93 (40.1)	12
Sex					
Men	614 (61.2)	79 (13.8)	244 (42.7)	249 (43.5)	42
Women	389 (38.8)	41 (10.9)	204 (54.3)	131 (34.8)	13
Race/ Ethnicity					
White	715 (71.3)	88 (13.1)	310 (46.3)	272 (40.6)	45
Black	112 (11.2)	12 (11.3)	54 (50.9)	40 (37.7)	6
Latino	117 (11.7)	18 (15.8)	53 (46.5)	43 (37.7)	3
Asian	31 (3.1)	0 (0.0)	14 (45.2)	17 (54.8)	0
Other race	28 (2.8)	2 (7.4)	17 (63.0)	8 (29.6)	1
Employed on 9/11					
No	28 (2.8)	6 (21.4)	12 (42.9)	10 (35.7)	0
Yes	974 (97.2)	114 (12.4)	436 (47.4)	369 (40.2)	55
Eligibility group					
Rescue and recovery worker	423 (42.2)	53 (13.8)	161 (41.8)	171 (44.4)	38
Date of arrival: 9/11/01^d^	324 (77.5)	41 (13.8)	122 (41.1)	134 (45.1)	27
Date of arrival: 9/12/01	34 (8.1)	3 (12.0)	12 (48.0)	10 (40.0)	9
Date of arrival: 9/13/01–9/17/01	30 (7.2)	7 (24.1)	11 (37.9)	11 (37.9)	1
Date of arrival: 9/18/01 and later	30 (7.2)	1 (3.4)	13 (44.8)	15 (51.7)	1
Total number of days worked: 1–7 days^d^	111 (27.2)	15 (13.9)	44 (40.7)	49 (45.4)	3
Total number of days worked: 7.5–30 days	100 (24.5)	15 (16.3)	36 (39.1)	41 (44.6)	8
Total number of days worked: 30.5–90 days	104 (25.5)	10 (11.5)	41 (47.1)	36 (41.4)	17
Total number of days worked: ≥90.5 days	93 (22.8)	11 (13.3)	35 (42.2)	37 (44.6)	10
Lower Manhattan resident	66 (6.6)	12 (18.5)	35 (53.8)	18 (27.7)	1
Lower Manhattan area worker/passerby	514 (51.3)	55 (11.0)	252 (50.6)	191 (38.4)	16
WTC exposure score					
None/low	25 (2.5)	5 (21.7)	15 (65.2)	3 (13.0)	2
Medium	228 (22.7)	45 (20.8)	107 (49.5)	64 (29.6)	12
High	491 (49.0)	55 (11.9)	216 (46.7)	192 (41.5)	28
Very high	259 (25.8)	15 (6.1)	110 (44.7)	121 (49.2)	13

^a^Includes only those with consistent reports of injury across Wave 1 and the HQoL Study

^b^Participants were asked specifically about their most serious injury received on 9/11

^c^Count of the following injury types: cut, broken bone, burn, concussion, sprain

^d^Among rescue/recovery workers, *date of arrival* and *number of days worked* refer to rescue and recovery work at the World Trade Center site

**Table 4:** Adjusted odds ratios (aOR) and 95% Confidence Intervals (CI) from multinomial logistic regression models for the associations between injury circumstances, demographic characteristics, eligibility groups, and World Trade Center (WTC) exposures and injury severity, Health and Quality of Life 15 Years after 9/11 Study

	Severity comparison	
	Medium vs. Low	High vs. Low
Characteristic	aOR^a^ (95% CI)	aOR^a^ (95% CI)
How injury occurred (among injured)^b^		
Hit by a falling object	0.65 (0.24, 1.74)	1.84 (0.70, 4.84)
Trip and fell	1.00 (Reference)	1.00 (Reference)
Hit head on object	0.33 (0.15, 0.72)	0.40 (0.17, 0.90)
Came into contact with something hot	1.12 (0.47, 2.69)	1.01 (0.41, 2.52)
Descending down stairs	1.00 (0.51, 1.95)	1.07 (0.54, 2.13)
Trapped, buried, or crushed	1.67 (0.45, 6.19)	1.80 (0.47, 6.87)
Covered by dust/debris/ash/asbestos	0.29 (0.05, 1.81)	1.08 (0.20, 5.87)
Unknown or unsure	0.17 (0.08, 0.34)	0.15 (0.07, 0.32)
Age at 9/11 (years)		
16–34	1.00 (Reference)	1.00 (Reference)
35–49	0.75 (0.39, 1.42)	0.71 (0.37, 1.37)
≥ 50	0.56 (0.28, 1.14)	0.70 (0.34, 1.44)
Sex		
Men	1.00 (Reference)	1.00 (Reference)
Women	1.66 (0.97, 2.86)	1.24 (0.71, 2.17)
Race/ Ethnicity		
White	1.00 (Reference)	1.00 (Reference)
Black	1.09 (0.50, 2.35)	1.23 (0.55, 2.74)
Latino	0.80 (0.42, 1.52)	0.85 (0.44, 1.67)
Asian^c^	3.48 (0.79, 15.25)	3.45 (0.77, 15.51)
Other race^c^	3.48 (0.79, 15.25)	3.45 (0.77, 15.51)
Employed on 9/11		
No	1.00 (Reference)	1.00 (Reference)
Yes	1.44 (0.41, 5.09)	0.85 (0.23, 3.18)
Eligibility group		
Rescue and recovery worker	1.44 (0.57, 3.65)	3.30 (1.22, 8.94)
Date of arrival: 9/11/01^d^	0.32 (0.04, 2.65)	0.23 (0.03, 1.87)
Date of arrival: 9/12/01	0.34 (0.03, 3.92)	0.22 (0.02, 2.55)
Date of arrival: 9/13/01–9/17/01	0.14 (0.01, 1.34)	0.11 (0.01, 1.11)
Date of arrival: 9/18/01 and later	1.00 (Reference)	1.00 (Reference)
Total number of days worked: 1–7 days^d^	1.00 (Reference)	1.00(Reference)
Total number of days worked: 7.5–30 days	0.90 (0.37, 2.17)	0.84 (0.35, 1.99)
Total number of days worked: 30.5–90 days	1.38 (0.53, 3.56)	0.95 (0.37, 2.47)
Total number of days worked: ≥90.5 days	1.07 (0.42, 2.74)	0.78 (0.31, 1.99)
Lower Manhattan resident	1.00 (Reference)	1.00 (Reference)
Lower Manhattan area worker/passerby	1.78 (0.73, 4.33)	3.43 (1.31, 8.97)
WTC exposure score		
None/low^e^	1.00 (Reference)	1.00 (Reference)
Medium^e^	1.00 (Reference)	1.00 (Reference)
High	1.94 (1.18, 3.17)	3.04 (1.81, 5.11)
Very high	4.05 (2.02, 8.11)	8.07 (3.95, 16.49)

^a^ Model controls for all factors listed in table, except for date of arrival and total number of days worked at the WTC site which applies to just rescue and recovery workers. A separate model was fit among rescue and recovery workers only that included these covariates as well. See footnote (d)

^b^ Participants were asked specifically about their most serious injury received on 9/11

^c^ Asian and other race were collapsed in the multinomial models due to small cell sizes

^d^ Among rescue/recovery workers, *date of arrival* and *number of days worked* refer to rescue and recovery work at the World Trade Center site. Corresponding odds ratios were generated from a separate model among rescue and recovery workers only

^e^ None/low and medium categories were collapsed in the multinomial models due to small cell sizes

Demographic characteristics and WTC-related activities and experiences were also related to injury severity in bivariate analyses. Men were more likely to experience high severity injuries compared with women (43.5% vs. 34.8%), and women were more likely to experience medium severity injuries compared with men (54.3% vs. 42.7%). Asians were the most likely to suffer high severity injuries compared with all other races and ethnicities (54.8% vs. 37.7% among Blacks and 40.6% among Whites). However, these apparent differences by sex and race/ethnicity were not statistically significant in multivariable analyses (Table 4). Rescue and recovery workers and lower Manhattan area workers were more likely to report high severity injuries (44.4%, aOR = 3.30, 95% CI, 1.22, 8.94, and 38.4%, aOR = 3.43, 95% CI, 1.31, 8.97, respectively) compared with lower Manhattan residents (27.7%). However, among rescue and recovery workers, the date of arrival to the WTC site or the total number of days worked at the site were unrelated to injury severity. The WTC exposure score, comprised of traumatic experiences on 9/11, was positively associated with injury severity, such that those with the highest scores were the most likely to have high severity injuries (49.2%, aOR = 8.07, 95% CI, 3.95, 16.49) compared with those with lower scores (13.0%).

Finally, the discrete number of injury types (i.e., cut, broken bone, burn, concussion, sprain) reported at Wave 1 was positively related to injury severity as measured on the HQoL Study questionnaire (Table 3). For example, 31.8% of those who reported one type of injury on 9/11 had high severity injuries compared with 78.6 and 100.0% of those with four and five injury types, respectively.

## Discussion

In a cohort of adults exposed to the 2001 World Trade Center attacks, an in-depth study on the long-term effects of injury was conducted 15 years after the disaster. This investigation served to document the process assessing the long-term health effects of injury more than a decade after the index event. Although participation rates were high overall (76%) and did not vary by initially reported injury status, several demographic characteristics such as age, education level, and race were predictors of participation. In addition, this study documented the extent to which participants had discrepant reporting on injury status over time. Approximately half of those who initially reported being injured directly after 9/11 reported on the follow-up study 15 years later that they had not been injured on 9/11. However, this discrepant reporting was most prevalent among those with few and minor injuries. Lastly, this study provided a comprehensive overview of the types of injuries sustained on 9/11 and characterized determinants of injury severity. Overall, most injuries occurred as a result of descending down stairs or by tripping and falling, whereas being hit by a falling object was associated with the most severe injuries.

Studying the long-term effects of injury may be difficult. As time since the index event of interest elapses, cohort attrition is common and participation in follow-up studies may become non-random. Furthermore, errors in recall about experiences related to the event of interest may arise or worsen over time. In this study, we have identified several factors related to study participation as well as discrepant reporting over time. These observations may aid researchers in their ability to draw conclusions about different study populations involved in research, as well as potentially inform future bias analyses.

Although participation rates did not vary by injury status, they varied by age, race/ethnicity, education, and self-rated health. Still, strata with the lowest rates still had a relatively high participation rate. For example, the participation rate for those aged 31–49 years was 69.8%; for those of other race was 68.1%; for those that had a high school education or less was 73.3%; and for those of poor health was 70.1%. This likely reflects, at least in part, that we drew our sample from those Registry enrollees who completed all four Waves. Participation rates across Waves were lower: 67.5% at Wave 2, 62.2% at Wave 3, and 52.8% at Wave 4. Still, selective participation across social and demographic strata has the potential to threaten internal validity through selection bias, and/or external validity (i.e., generalizability) (Goldberg et al. 2001; Kramer et al. 2009).

The prevalence of discrepant reports, especially among those who reported that they were injured at Wave 1, was high. However, we found that those who reported more minor injuries at Wave 1 (i.e., a sprain versus a broken bone or concussion; or just one injury versus two or more injury types) were the ones who were most likely to report *not* being injured at follow-up on the HQoL Study questionnaire. This suggests that these discrepancies in reporting among the injured group were due mostly to recall error, making our sample who consistently reported that they were injured (*n* = 1003) more severely injured overall than all those who reported injury soon after 9/11 at Wave 1 (*n* = 2699). This phenomenon of severe conditions being more likely to be recalled has been documented previously in the literature (Beckett et al. 2000, 2001). In a longitudinal study on health conditions among Taiwanese adults, the primary predictor of recall over time was the initial severity of the condition, and the degree to which it limited activities or caused inconveniences in daily living (Beckett et al. 2000).

This evidence of differential discrepant reporting based on severity has the potential to affect both the internal and external validity of studies that rely exclusively on retrospectively recalled information. For example, if injury were the exposure of interest in a hypothetical study, and information shortly after the event was not available, information bias could result from those participants who misreport their injury status. This could be differential or non-differential with respect to the outcome, depending on the particular outcome under study. However, in our illustrative analysis examining the potential association with PTSD at Wave 4, estimates did not greatly differ when we ran models in which one used injury on 9/11 status from Wave 1 and another used injury on 9/11 status from the HQoL study. In addition, recall error could also affect the external validity of studies that rely on recall of injury status due to the relative exclusion of those with more minor injuries, resulting a sample with more severe injuries overall. Therefore, this could limit generalizability to more severely injured populations overall.

In addition, WTC-related exposures and PTSD history were associated with increased discrepant reporting both among those who reported that they were and were not injured at Wave 1. Among those who initially reported not being injured, those who had a high degree of WTC exposures or ever had PTSD were more likely to change their response from ‘not injured’ at Wave 1 to ‘injured’ on the HQoL Study. These associations suggest that these individuals have traumatizing memories of 9/11 and thus may have apportioned additional suffering to that experience (Roemer et al. 1998). This is consistent with the corresponding observations among those who reported being injured at Wave 1: high levels of WTC exposures and PTSD were associated with a *decreased* likelihood of discrepant reporting (i.e., consistent reporting of injury). However, it is also possible that their psychological experience since 9/11 may have heightened their memories of what occurred on 9/11 (Weber 2008). Although discrepant reports were rare among those who initially said they were not injured (7.3%), this type of differential recall error could be another source of bias in epidemiologic studies where the exposure or outcome of interest is injury status.

A previous Registry study provided an overall estimate of injury among enrollees of 13%, with rescue and recovery workers (15.2%) and building occupants or passersby (15.9%) being the most likely to incur an injury and students the least likely (3.8%) (Farfel et al. 2008). Another Registry study specifically focused on survivors of collapsed or damaged buildings reported that 43.6% sustained an injury, and found the dust/debris cloud, building type, evacuation time, and floor occupancy to be associated with the likelihood of injury (Brackbill et al. 2006). However, these early studies did not have information on injury severity, medical treatments sought after the injury, or how the injuries were sustained. Through the continued follow-up of this cohort and the HQoL Study, this study addresses these important gaps.

This study benefited from long-term follow-up on a well-defined cohort formed shortly after the index exposure of interest (9/11). For one, this allowed us to compare short-term recall with long-term recall of injury status. In addition, this cohort includes both those who were rescue and recovery workers on 9/11 as well as those who lived and worked in lower Manhattan at the time. Inclusion of these heterogeneous groups allows for a better understanding of the total impact of injury in the affected population. Lastly, the HQoL study included questions on how injury occurred, and what types of medical intervention were sought after the injury was sustained. This is important data that have seldom been collected in 9/11-exposed populations (Berríos-Torres et al. 2003).

However, this study was also subject to some limitations. First, although our study was able to identify factors associated with participation, the generalizability of these findings may be limited because those eligible for this study were restricted to an already highly compliant portion of the Registry cohort: those that had previously completed all four of the major survey waves (Waves 1 through 4). This is an important caveat to consider, as other studies may not recruit from this same type of sub-cohort. Furthermore, these compliant enrollees who completed multiple waves were different than those who dropped out by several sociodemographic characteristics (Yu et al. 2015). For example, non-White enrollees were less likely to complete all four waves compared with White enrollees: non-White enrollees comprised 37.0% of the total cohort at Wave 1 vs. 26.7% of those who completed all four Waves. However, injury status as specified at Wave 1 did not seem to be related to longitudinal participation in survey waves: injured enrollees comprised 17.5% of the total cohort at Wave 1 and 18.3% of those who completed all four Waves. Another limitation was that all of our data was self-reported. For example, we relied on self-report of medical intervention after injury, which is likely not as accurate as clinical information or data from medical records would have been to inform injury severity. Furthermore, we treated the Wave 1 report of injury status as the ‘gold standard’ in our analysis of discrepant reporting over time. Because Wave 1 was administered 2–3 years after 9/11, it is possible that this information was subject to recall error, in addition to the later reports on the HQoL questionnaire. In an ideal setting, objective data would have been obtained. Finally, our multinomial analysis of injury severity resulted in estimates with poor precision (i.e., wide confidence intervals). Still, we included this analysis to convey relative determinants of injury severity across the distribution of severity (i.e., low, medium, and high). Furthermore, it is important to note that this analysis and study implicitly excluded those whose injuries resulted in death. Although the target population in this study are 9/11 survivors, including these injuries could be of interest as they were likely the most severe. Despite these limitations, this study took advantage of the rich information and the longitudinal design over 15 years of follow-up on a diverse cohort of individuals exposed to the 9/11 terrorist attacks.

## Conclusions

This study serves as a report of the issues related to conducting a follow-up study on injury more than a decade after the initial incident. Although participation rates were high overall and did not vary by injury status, demographic factors were related to study participation. Consistent recall of injury over time was much less prevalent among those who initially reported minor injuries compared to those who reported more severe injuries as well as those who reported not being injured at all. Finally, most injuries occurred while descending down stairs or by tripping and falling, whereas being hit by a falling object was associated with the most severe injuries. This study provides important descriptive information that may be useful for future studies investigating the impacts of injury over time.

## Additional file

Additional file 1: **Table S1.** Injury on 9/11 status by demographics, World Trade Center exposures, and posttraumatic stress disorder history among those who reported injury status consistently over time. **Table S2.** Associations between injury on 9/11 as assessed at different time points and posttraumatic stress disorder (PTSD) statusa at World Trade Center Health Registry Wave 4. (DOCX 17 kb)

## Abbreviations

9/11: September 11, 2001
aRR: Adjusted risk ratio
CI: 95% confidence interval
HQoL Study: The Health and Quality of Life 15 Years after 9/11 Study
PCL: Posttraumatic disorder checklist
PTSD: Posttraumatic disorder
WTC: World Trade Center
