# Supplementary material for: Conducting a study to assess the long-term impacts of injury after 9/11: participation, recall, and description
Source: Inj Epidemiol. 2019 Mar 18;6:8. doi: 10.1186/s40621-019-0186-y (PMC6582679; doi:10.1186/s40621-019-0186-y)
Supplement: Supplementary file 1 — Table S1. Injury on 9/11 status by demographics, World Trade Center exposures, and posttraumatic stress disorder history among those who reported injury status consistently over time. Table S2. Associations between injury on 9/11 as assessed at different time points and posttraumatic stress disorder (PTSD) statusa at World Trade Center Health Registry Wave 4. (DOCX 17 kb) [file 40621_2019_186_MOESM1_ESM.docx]

| **Supplemental Table 1**. Injury on 9/11 status by demographics, World Trade Center exposures, and posttraumatic stress disorder history among those who reported injury status consistently over time | | | | | | |  |
| --- | --- | --- | --- | --- | --- | --- | --- |
|  | Total  (N=2,821, 100%) | | Injured (N=1,003, 35.6%) | | Non-injured (N=1,818, 64.4%) | |  |
|  | N | % | N | % | N | % |  |
| Age at 9/11 (years) |  |  |  |  |  |  |  |
| 16-34 | 660 | 23.4 | 181 | 18.0 | 479 | 26.3 |  |
| 35-49 | 1395 | 49.5 | 578 | 57.6 | 817 | 44.9 |  |
| ≥50 | 766 | 27.2 | 244 | 24.3 | 522 | 28.7 |  |
| Sex |  |  |  |  |  |  |  |
| Male | 1589 | 56.3 | 614 | 61.2 | 975 | 53.6 |  |
| Female | 1232 | 43.7 | 389 | 38.8 | 843 | 46.4 |  |
| Race/ Ethnicity |  |  |  |  |  |  |  |
| White | 2101 | 74.5 | 715 | 71.3 | 1386 | 76.2 |  |
| Black | 267 | 9.5 | 112 | 11.2 | 155 | 8.5 |  |
| Hispanic | 267 | 9.5 | 117 | 11.7 | 150 | 8.3 |  |
| Asian | 109 | 3.9 | 31 | 3.1 | 78 | 4.3 |  |
| Other race | 77 | 2.7 | 28 | 2.8 | 49 | 2.7 |  |
| Education at Wave 1 |  |  |  |  |  |  |  |
| ≤High school/ GED | 421 | 15.0 | 213 | 21.3 | 208 | 11.5 |  |
| Some college | 585 | 20.8 | 290 | 29.0 | 295 | 16.3 |  |
| College | 1081 | 38.4 | 327 | 32.7 | 754 | 41.5 |  |
| Graduate degree | 728 | 25.9 | 170 | 17.0 | 558 | 30.7 |  |
| Marital Status at Wave 1 |  |  |  |  |  |  |  |
| Married/cohabitating | 1818 | 64.7 | 645 | 64.4 | 1173 | 64.8 |  |
| Divorced | 303 | 10.8 | 149 | 14.9 | 154 | 8.5 |  |
| Widowed | 63 | 2.2 | 31 | 3.1 | 32 | 1.8 |  |
| Never married | 627 | 22.3 | 176 | 17.6 | 451 | 24.9 |  |
| Income at Wave 1 |  |  |  |  |  |  |  |
| ≤$50,000 | 552 | 21.6 | 247 | 27.0 | 305 | 18.6 |  |
| $50,000-$150,000 | 1564 | 61.2 | 559 | 61.2 | 1005 | 61.2 |  |
| ≥$150,000 | 441 | 17.2 | 108 | 11.8 | 333 | 20.3 |  |
| Employed on 9/11 |  |  |  |  |  |  |  |
| No | 113 | 4.0 | 28 | 2.8 | 85 | 4.7 |  |
| Yes | 2704 | 96.0 | 974 | 97.2 | 1730 | 95.3 |  |
| Eligibility group |  |  |  |  |  |  |  |
| Rescue and recovery worker | 664 | 23.5 | 423 | 42.2 | 241 | 13.3 |  |
| Lower Manhattan resident | 318 | 11.3 | 66 | 6.6 | 252 | 13.9 |  |
| Lower Manhattan area worker/passerby | 1839 | 65.2 | 514 | 51.2 | 1325 | 72.9 |  |
| WTC exposure score |  |  |  |  |  |  |  |
| None/low | 542 | 19.2 | 5 | 0.5 | 537 | 29.5 |  |
| Medium | 987 | 35.0 | 91 | 9.1 | 896 | 49.3 |  |
| High | 757 | 26.8 | 402 | 40.1 | 355 | 19.5 |  |
| Very high | 535 | 19.0 | 505 | 50.3 | 30 | 1.7 |  |
| Ever-PTSD^a^ |  |  |  |  |  |  |  |
| No | 1671 | 62.7 | 314 | 32.9 | 1357 | 79.3 |  |
| Yes | 995 | 37.3 | 641 | 67.1 | 354 | 20.7 |  |
| ^a^Ever had a PCL score ≥ 44 on Wave 1, Wave 2, Wave 3, and/or Wave 4 | | | | | | |  |

| **Supplemental Table 2.** Associations between injury on 9/11 as assessed at different time points and posttraumatic stress disorder (PTSD) status^a^ at World Trade Center Health Registry Wave 4 | | | |
| --- | --- | --- | --- |
| Exposure comparison | N (%) with PTSD among injured | N (%) with PTSD among non-injured | aRR^b^ (95% CI) |
| Reported injury at Wave 1 vs. did not^c^ | 521 (27.1%) | 150 (7.8%) | 3.38 (2.83, 4.02) |
| Reported injury at HQoL study vs. did not^d^ | 377 (35.1%) | 283 (10.4%) | 3.20 (2.78, 3.69) |
| Reported injury at both time points vs. reported no injury at both time points | 344 (36.4%) | 113 (6.4%) | 5.71 (4.66, 7.00) |

^a^PCL score ≥ 44 on Wave 4

^b^Adjusted for age at 9/11, sex, race/ethnicity, employment status on 9/11, and WTCHR eligibility group

^c^Among those who also completed the HQoL study

^d^N=82 were missing injury status on the HQoL study
